# Mitochondrial Dysfunction in Sporadic Amyotrophic Lateral Sclerosis Patients: Insights from High-Resolution Respirometry

**DOI:** 10.3390/biomedicines12061294

**Published:** 2024-06-11

**Authors:** Petra Parvanovova, Andrea Evinova, Milan Grofik, Petra Hnilicova, Zuzana Tatarkova, Monika Turcanova-Koprusakova

**Affiliations:** 1 Department of Medical Biochemistry, Jessenius Faculty of Medicine, Comenius University in Bratislava, 036 01 Martin, Slovakia; parvanovova1@uniba.sk (P.P.); zuzana.tatarkova@uniba.sk (Z.T.); 2 Biomedical Centre Martin, Jessenius Faculty of Medicine, Comenius University in Bratislava, 036 01 Martin, Slovakia; andrea.evinova@uniba.sk (A.E.); petra.hnilicova@uniba.sk (P.H.); 3 Department of Neurology, University Hospital Martin, 036 01 Martin, Slovakia; milan.grofik@uniba.sk

**Keywords:** amyotrophic lateral sclerosis, mitochondria, high-resolution respirometry, mitochondrion dysfunction, complex I, complex II

## Abstract

Amyotrophic lateral sclerosis is a severe neurodegenerative disease whose exact cause is still unclear. Currently, research attention is turning to the mitochondrion as a critical organelle of energy metabolism. Current knowledge is sufficient to confirm the involvement of the mitochondria in the pathophysiology of the disease, since the mitochondria are involved in many processes in the cell; however, the exact mechanism of involvement is still unclear. We used peripheral blood mononuclear cells isolated from whole fresh blood from patients with amyotrophic lateral sclerosis for measurement and matched an age- and sex-matched set of healthy subjects. The group of patients consisted of patients examined and diagnosed at the neurological clinic of the University Hospital Martin. The set of controls consisted of healthy individuals who were actively searched, and controls were selected on the basis of age and sex. The group consisted of 26 patients with sporadic forms of ALS (13 women, 13 men), diagnosed based on the definitive criteria of El Escorial. The average age of patients was 54 years, and the average age of healthy controls was 56 years. We used a high-resolution O2K respirometry method, Oxygraph-2k, to measure mitochondrial respiration. Basal respiration was lower in patients by 29.48%, pyruvate-stimulated respiration (respiratory chain complex I) was lower by 29.26%, and maximal respiratory capacity was lower by 28.15%. The decrease in succinate-stimulated respiration (respiratory chain complex II) was 26.91%. Our data confirm changes in mitochondrial respiration in ALS patients, manifested by the reduced function of complex I and complex II of the respiratory chain. These defects are severe enough to confirm this disease’s hypothesized mitochondrial damage. Therefore, research interest in the future should be directed towards a deeper understanding of the involvement of mitochondria and respiratory complexes in the pathophysiology of the disease. This understanding could develop new biomarkers in diagnostics and subsequent therapeutic interventions.

## 1. Introduction

Amyotrophic sclerosis is a fatal neurodegenerative disease with a median survival of 3 to 5 years [1]. The disease is characterized by the progressive neurodegeneration of motor neurons in the brain and spinal cord at all levels of the motor system. The prevalence of this disease is approximately 6/100,000 persons [2]. Even nowadays, there is no evidence to fully elucidate the pathophysiology of this severe disease. Possible theories deal with the influence of genetics and environmental factors, but the involvement of multiple factors in developing the disease is not excluded. The literature also describes possible mechanisms such as genetic mutations, oxidative stress, excitotoxicity, mitochondrial and proteasomal dysfunction, impaired axonal transport, and neuroinflammation [3]. Table 1 provides a more detailed overview. A thorough understanding of the pathophysiology of the disease is a crucial basis for the subsequent development of effective therapeutic interventions. The unidirectional focus on possible pathophysiology may account for the unsatisfactory outcomes of potential therapeutic approaches.

The clinical symptoms of the disease can be divided according to the affected area/level into bulbar and cervical or lumbar forms (spinal form of the disease) [22]. The onset of the bulbar form of the disease is usually represented by slurred speech, difficulty chewing and swallowing, excessive choking, and weakness or twitching of the muscles of the face, jaw, throat, and vocal cords, especially the tongue. The spinal form of the disease is characterized by symptoms associated with focal muscle weakness and flaccidity, the onset of which may be distal or proximal in the upper and lower limbs. As the damage progresses, spasticity develops in the weakened atrophic limbs. There is also a known respiratory form of the disease, where respiratory symptoms are the first to appear without more severe damage to the limbs or bulbs. Common symptoms of this form of ALS include nocturnal hypoventilation, dyspnoea, orthopnoea, sleep disturbances, morning headaches, excessive daytime somnolence, anorexia, reduced concentration, and irritability or mood changes [23]. This form of the disease affects approximately 5% of patients [24]. The most common cause of death in patients is respiratory muscle paralysis and subsequent respiratory failure.

Diagnosis of this disease is also challenging, involving clinical assessment of the patient’s condition and exclusion of other potentially treatable diseases. The average diagnostic delay for this disease is 10 to 16 months [25]. Diagnosis of the disease is primarily clinical and relies on El Escorial criteria. The diagnostic procedure consists of obtaining a detailed history, conducting a clinical examination, and using electrodiagnostic, imaging, and laboratory methods. A thorough family history, especially the presence of motor neuron diseases, should be considered. As part of the examination, we observe symptoms in people with bulbar ALS related to speech and swallowing, while the first symptoms in people with limb or spinal ALS are related to the limbs and trunk [26]. The gold standard in assessing patient status and rate of progression is currently the ALS Functional Rating Scale (ALSFRS) and its revised form (ALSFRS-R). This questionnaire includes questions targeting bulbar function, fine and gross motor tasks, and respiratory function. However, more advanced staging systems are now available (King’s Staging System, Milano Torino Staging System, Rasch Overall ALS Disability Scale) [1]. Nerve conduction studies are required as part of electrodiagnostic methods to demonstrate motor nerve involvement, indicated by reduced action potential of the compound muscle, prolonged distal motor latency, and reduced conduction velocity without evidence of conduction blocks. Another method is needle EMG, which is necessary for assessing acute and chronic denervation in ALS patients. Muscle ultrasonography can increase diagnostic certainty in this disease due to its excellent sensitivity for detecting fasciculations. A relatively new approach is nerve ultrasonography, which has shown smaller nerves and nerve roots in ALS patients; it also has the potential to differentiate from multifocal motor neuropathy [27]. Classical MRI is used to rule out other diseases. Still, an interesting method is MRI sequence assessment of functional connectivity to determine impairment of the peripheral and central motor nervous system in ALS [28]. Another method is genetic testing, which can find confirmed genetic variants and rule out other genetic disorders. Within laboratory diagnostics, potentially promising biomarkers in diagnosing this disease are neurofilaments, inflammatory biomarkers, Chitinase, Tau Protein, TDP-43, and Creatine Kinase [1]. There are currently three drugs approved for the treatment of ALS (Riluzole and Edavarone, Sodium phenylbutyrate, and Taurursodiol PB/TURSO), which do not cure the disease but help slow the progression of this serious disease. 

Mitochondrial dysfunction in ALS patients has been known for more than 50 years, and the involvement of mitochondria in the pathophysiology of this disease is now widely accepted [29]. Motor neurons are particularly vulnerable to mitochondrial malfunctions due to high energy demands. However, it is not known whether mitochondria have a role as a primary trigger in the pathophysiology of the disease or if this is a consequence of other cellular processes. Mitochondrial dysfunction has been demonstrated in ALS patients as well as in experimental models in vivo and in vitro. Abnormal mitochondrial morphology, increased reactive oxygen species production, defects in mitochondrial dynamics, altered respiratory chain enzyme activities, and impaired Ca2 homeostasis have been described [30]. When mitochondrial homeostasis is disrupted, not only oxidative phosphorylation is reduced but the mitochondrial respiratory chain is disrupted. This can further damage mitochondria and lead to the release of mitochondrial proapoptotic proteins such as cytochrome c and apoptosis-inducing factor, ultimately leading to programmed cell death [31]. A comprehensive view of the role of mitochondria and its involvement in the pathomechanisms of this disease may contribute to the development of new diagnostic and therapeutic strategies.

Our study aimed to determine mitochondrial respiration in 26 patients with amyotrophic lateral sclerosis (13 males and 13 females) and subsequent comparison with healthy subjects, correlated based on age and sex.

## 2. Materials and Methods

### 2.1. Patient Cohort

The study population consisted of patients examined and diagnosed at the Department of Neurology, University Hospital Martin, Martin, Slovakia, between June 2021 and February 2023. The study population included 26 subjects (13 males and 13 females, the mean age was 54 years, and the mean ALSFR-S score at the time of diagnosis was 35.11 points. The mean diagnostic delay was 12.26 months. Twenty-one patients developed the spinal form of the disease, and five patients developed the bulbar form of the disease. The oldest patient was 80 years old, and the youngest was 28 years old. All the patients were Caucasian. Patients were diagnosed according to El Escorial criteria, which were satisfied by all patients. EMG findings in all patients met the criteria for diagnosing ALS (according to Awai Shima criteria) [32,33]. Inclusion and exclusion criteria for patients are summarized in Table 2.

Some patients also had comorbidities: (1) cardiovascular comorbidities, the most common of which were arterial hypertension (12 patients), ischemic heart disease (2 patients), myocardial infarction (1 patient), and cardiac autonomic neuropathy (1 patient); (2) respiratory comorbidities: bronchial asthma (3 patients), chronic obstructive pulmonary disease (1 patient), and spastic bronchitis (1 patient); (3) liver comorbidities: hepatic steatosis (3 patients), hepatopathy (2 patients), and mild hepatomegaly (1 patient); (4) thyroid comorbidities: Graves–Basedow disease (1 patient), Sjogren’s syndrome (1 patient), thyroiditis (1 patient), and thyroidopathy (1 patient); (5) metabolic comorbidities: hyperlipidemia (5 patients), diabetes mellitus (3 patients), hyperuricemia (3 patients), and hyperbilirubinemia (1 patient); (6) autoimmune comorbidities: rheumatoid arthritis (1 patient); (7) neurologic diseases: epilepsy (2 patients); and (8) other comorbidities: lyme disease (2 patients), anemia (1 patient), hypovitaminosis vit. D (1 patient). Detailed characteristics of the patient cohort are available in Table 3.

The control group was matched based on a correlation of age and sex with no symptoms or history of neurological disease. The control group consisted of 13 males and 13 females; the mean age was 56. The inclusion and exclusion criteria for the control group are summarized in Table 4.

Written informed consent was obtained from all patients before the examination. The informed consent process followed ethical guidelines and ensured participants were well-informed and voluntarily participated in the research protocol. The research project was approved by the ethics committee under the number EC 43/2021. A 10 mL quantity of venous blood was collected through a single venipuncture. EDTA tubes were used for the collection. The blood was transported to the laboratory in the shortest possible time and then immediately processed and analyzed. Isolation of PBMCs was performed within a maximum of 1 hour after collection.

### 2.2. Peripheral Blood Mononuclear Cell Isolation

PBMCs (peripheral blood mononuclear cells) isolated from fresh venous blood were used for measurement. Fresh peripheral venous blood samples were collected into EDTA-containing tubes. A commercially available Leucosep kit (Greiner bio-one GmbH, Kremsmünster, Austria) was used to isolate PBMCs followed by post-treatment according to the instructions for use. Histopaque-1077 (Sigma-Aldrich, St. Louis, MO, USA) warmed at room temperature was used as the separation medium. Subsequently, the buffy coat was gently aspirated and washed twice with DPBS.

Further details of the methodology adopted can be found in Sumbalova et al. [34]. Subsequently, the washed PBMC pellet was dispersed in 250 µL of MiR05 respiration medium (Oroboros Instruments GmbH, Innsbruck, Austria), and 100 µL of cell suspension was injected into each 2 mL glass chamber.

### 2.3. High-Resolution Respirometry

Mitochondrial function was determined by high-resolution O2K respirometry, Oxygraph-2k (Oroboros Instruments GmbH, Innsbruck, AT, Austria). Measurements were performed in a two-chamber system at 37 °C with a stirring speed of 750 rpm. The amplified signal from the oxygen sensor was recorded by computer with scanning intervals of 2 s using DatLab 7 software (Oroboros Instruments, Innsbruck, AT). Before starting all experiments, the respirometer was calibrated at air saturation and 37 °C. Manual titration of inhibitors and uncouplers was performed using Hamilton syringes. A standard coupling control protocol (CCP) was used to determine mitochondrial function. All aliquots of substrates and inhibitors were stored at −20 °C. Pyruvate was prepared fresh daily. Following the Oroboros guidelines [35], the resulting O2 flux values were used to calculate respiration in different states of coupling control, including basal respiration (ROUTINE), LEAK, maximal electron transport (ET) capacity, respiratory reserve, ATP-coupled respiration, and succinate-driven stimulation. The measurements allowed analysis of the main parameters of mitochondrial respiration: ROUTINE (R) respiration, which represents physiological cell respiration without being affected and is measured after titration of the cell suspension into chambers containing mitochondrial respiration medium; pyruvate-stimulated respiration (P, 10 mM), which is measured after addition of P as substrate for I. in intact cells; oligomycin (Omy, 10 nM), which is used to induce the LEAK respiratory state; maximal ET capacity, which represents the attainment of maximal respiration by sequential titration of Uncoupler (U*-CCCP, 0.5 µM sequential titration); Rotenone (Rot, 500 nM) as a complex I (CI) inhibitor, which is used for ROX; Succinate (S, 10 mM) stimulation of mitochondrial respiration, which is only observed in nonliving/permeabilized cells; digitonin (Dig, 10 mg/mL sequential addition), which is used to permeabilize the entire population of cells, providing a reference state for succinate-stimulated ETS capacity; cytochrome C titration assay (c, 10 µM), which provides information on the integrity of the outer mitochondrial membrane; and antimycin A (Ama, 2.5 µM), which is a complex III inhibitor. Residual oxygen consumption (ROX) is assessed to correct for the flow of intact cellular respiration, and ROX is respiration that is associated with other oxygen consumption processes in the cell in addition to the respiratory system.

### 2.4. Determination of Protein Concentration

Protein concentration determinations were performed to determine the exact amount of mitochondria. Protein concentration was determined using the DC Protein assay (BioRad, Hercules, CA, USA). The first step was centrifugation at 1000× *g* for 10 min, followed by aspiration without disturbing the visible protein pellet. The pellet was then dispersed and adequately mixed in 200 microliters of RIPA analysis and extraction buffer (Thermo Scientific, Waltham, MA, USA). This was followed by a 30-min incubation at 4 °C, during which protein lysis occurred. This was followed by centrifugation at 14,000× *g* for 15 min at 4 °C. A 96-well plate was used to measure the proteins, and 5 microliters of analyte and 5 microliters of standards were pipetted with increasing concentrations from 0.125 mg/mL to 2 mg/mL. Pre-diluted protein assay standards were used for standard preparation: BSA (bovine serum albumin) Set (Thermo Scientific). Subsequently, 25 microliters of reagent A (basic copper tartrate solution) and 200 microliters of reagent B (dilute folin reagent) were added to each well. The plate was incubated for 15 min in the dark. The absorbance was measured on a Synergy H4 instrument (BioRad, Hercules, CA, USA) at 750 nm. The protein concentration in milligrams was calculated based on the calibration curve of individual absorbance.

### 2.5. Statistical Analysis

Statistical analysis was performed using PRISM GraphPad version 9.1.2 (La Jolla, CA, USA). All figures were also created in this program. The unpaired (Mann–Whitney) *t*-test was used in the statistical analysis. Significance values were set at *p* > 0.05, * *p* ≤ 0.05, ** *p* ≤ 0.01, *** *p* ≤ 0.001, and **** *p* ≤ 0.0001. Spearman’s test of correlation was also used.

## 3. Results

**Figure 1:**
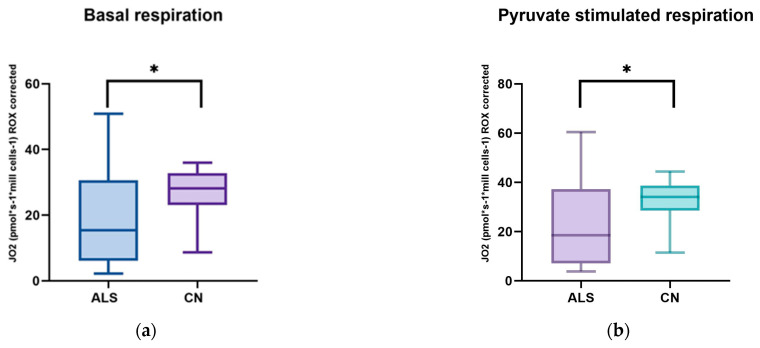

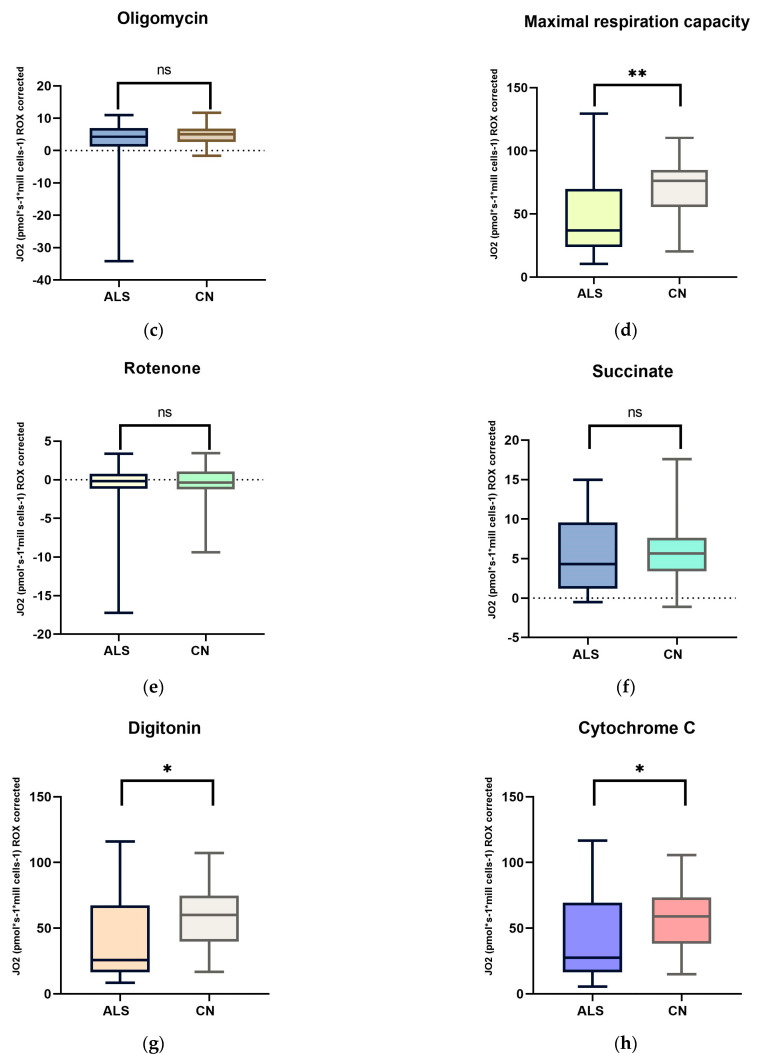

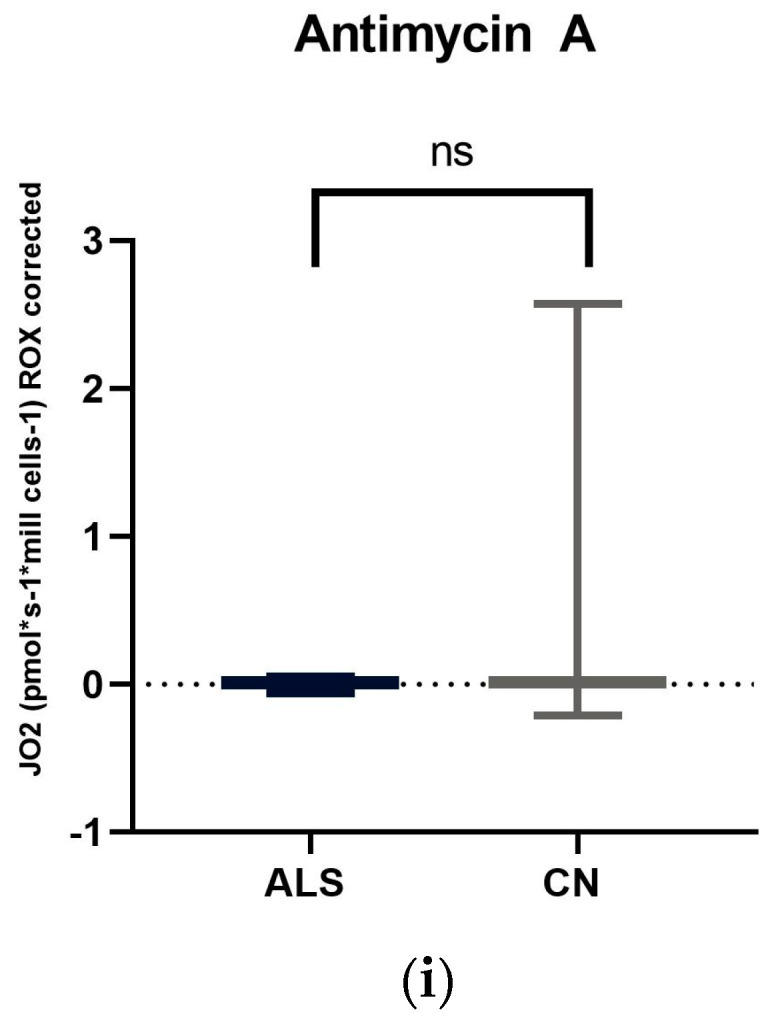
Comparison of mitochondrial respiration in patients with amyotrophic lateral sclerosis and healthy subjects: (**a**) basal respiration significant difference *p* = 0.0123; (**b**) pyruvate −stimulated respiration significant difference *p* = 0.011; (**c**) respiration after addition of oligomycin, no significant difference *p* = 0.4843; (**d**) maximal respiratory capacity, significant difference *p* = 0.0059; (**e**) respiration after addition of rotenone, no significant difference *p* = 0.817; (**f**) respiration after stimulation with succinate without significant differences *p* = 0.5673; (**g**) respiration after addition of digitonin, significant difference *p* = 0.0144; (**h**) respiration after addition of cytochrome C significant difference *p* = 0.0253; and (**i**) respiration after addition of Antimycin A without significant differences *p* = 0.1604. ns: *p* > 0.05; * *p* ≤ 0.05; ** *p* ≤ 0.01.

**Figure 2:**
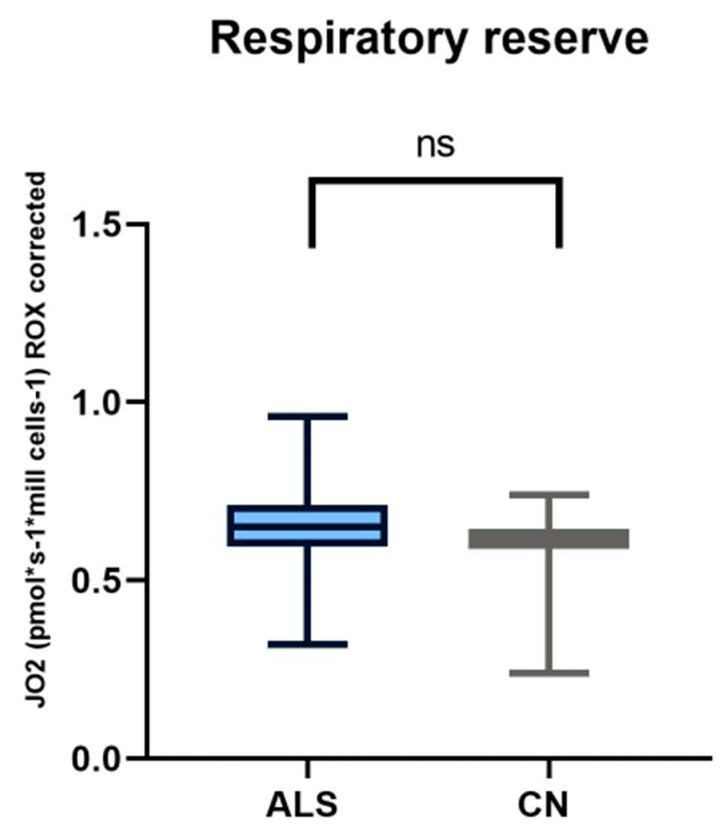
In comparing respiratory reserve in patients with amyotrophic lateral sclerosis and healthy controls, we observed no significant differences.

We focused on measuring mitochondrial respiration in ALS patients against sex- and age-matched controls. Our cohort consisted of 26 patients. We used a high-resolution respirometry method, Oroboros (AT), for the measurement. After adding intact cells, we observed basal respiration, i.e., energy demand under steady-state conditions. Subsequently, we added pyruvate, which is the substrate for complex I. After the addition of oligomycin, ATPase was inhibited. Subsequently, we measured the maximal respiratory capacity using sequential uncoupler titration. With the addition of rotenone, complex I was inhibited. Subsequently, we added succinate, the substrate for the second complex. With the gradual addition of digitonin, permeabilization of the plasma membrane occurs, and mitochondrial respiration (dependent on complex II) increases until complete permeabilization, without disruption of the inner cell membrane, where succinate and ADP enter the cells. Cytochrome C was added to test the integrity of the outer mitochondrial membrane. The final step was the addition of Antimicin A, which serves to inhibit complex III. The mean respiratory values of ALS patients and healthy controls are shown in Table 5 and Figure 1 and Figure 2.

We observed significant changes between ALS patients and healthy controls in basal respiration (*p* = 0.0123), pyruvate-stimulated respiration (*p* = 0.011), and maximal respiratory capacity (*p* = 0.0059), changes were also observed in succinate-stimulated respiration, after the addition of digitonin (*p* = 0.0144) and Cytochrome C (*p* = 0.0253).

**Figure 3:**
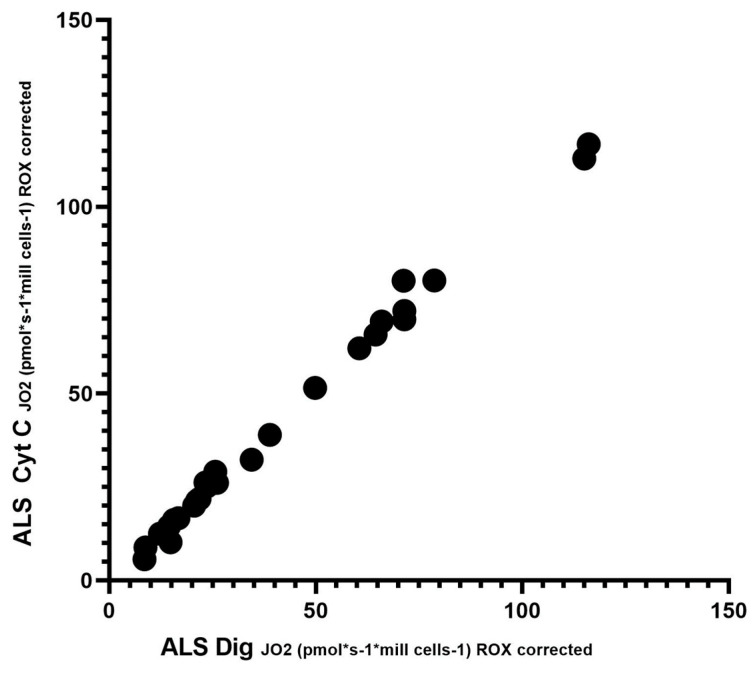
Correlation between digitonin and cytochrome C; Spearman’s correlation test.

To assess significant differences in respiration after the addition of digitonin and cytochrome C, Spearman’s correlation test was performed. Since respiration after adding cytochrome C should not increase complex II-dependent respiration, this test demonstrated no loss of cytochrome C from the outer mitochondrial membrane, and mitochondrial integrity is preserved. This test showed a significantly high correlation (r = 0.9925; *p* < 0.0001). Spearman’s correlation is shown in Figure 3. 

In our cohort of patients, we observed significant differences in basal respiration pyruvate-stimulated respiration, which is indicative of malfunctioning of complex I. Furthermore, maximal respiratory capacity was significantly reduced in patients. We also observed significant differences after adding digitonin and Cytochrome C (succinate stimulated respiration), indicating malfunctioning of complex II. We then used Spearman’s correlation test, which showed a linear relationship between respiration after adding digitonin and Cytochrome C.

## 4. Discussion

Amyotrophic lateral sclerosis is a severe and incurable neurodegenerative disease. Despite considerable research efforts, its pathophysiology is unclear. We aimed to determine mitochondrial respiration in ALS patients and sex- and age-correlated controls. We observed a decrease in basal respiration and respiration after pyruvate stimulation, indicating reduced complex I function. Maximal respiratory capacity was also significantly lower in patients. Respiration after adding digitonin (succinate-stimulated respiration) was also significantly lower in patients, indicating decreased complex II function.

The mitochondria is a highly dynamic organelle crucial in cell survival and for maintaining normal metabolism, energy homeostasis, calcium homeostasis, cell death mechanisms, redox balance, cell growth, and differentiation [36]. It is also involved in antioxidant metabolism and axonal transport [37]. Mitochondria are abundantly represented and actively transported in regions of neurons with intense demands for energy in the form of ATP, such as axon hillocks, nodes of Ranvier, and synaptic regions. Their subcellular composition can change dynamically according to the physiological needs of the cell [38]. However, the damage and subsequent malfunction of mitochondria results in a decrease in ATP production, leading to neuronal damage. 

Mitochondrial damage also mediates intraneuronal damage and death of motor neurons through calcium-mediated excitotoxicity, activation of the intrinsic apoptotic pathway, and increased ROS production [39,40,41,42]. The progressive changes in mitochondrial morphology, bioenergetics, and calcium homeostasis have been linked to pathological changes in ALS. Furthermore, many genes (*SOD1*, *FUS*, *VAPB*, *TARDBP*, *OPTN*, *VCP*, *C9orf72*) shown to be involved in the pathophysiology of the disease are closely linked to mitochondria and their function [43]. In studies of disease models in vitro [44,45] and animal models [46,47,48,49], mitochondrial damage was also observed. Last but not least, this damage has also been demonstrated by studies on patients or in muscles [50,51,52,53], peripheral blood [54,55,56,57], and spinal cord [58]. 

In our work, we used PBMCs to investigate the changes; the main advantage is their easy accessibility, as they are isolated from blood. Therefore, monitoring their abnormalities could be a useful prognostic and diagnostic marker of ALS. Araujo et al. also used PBMCs to look at mitochondrial damage in ALS. Their experiments led to the following conclusions: lower mitochondrial calcium uptake/retention, mitochondrial depolarization, and impaired redox homeostasis. They also showed a decrease in biogenesis and mitochondrial number and a decrease in energy-producing metabolic compounds such as ATP and pyruvate production in the PBMC cells studied [59].

We used a high-resolution respirometry method to analyze mitochondrial respiration. This method is probably the most rigorous procedure to assess the state of mitochondrial respiration, by adding specific electron-providing substrates or inhibitors of individual complexes [60]. In ALS patients, we observed significantly lower basal respiration, pyruvate-stimulated respiration, maximal respiratory capacity, and also a decrease in respiration after the addition of digitonin compared to healthy controls. Respiratory reserve did not show significant changes compared to healthy controls. Basal respiration represents the oxygen consumption rate in ATP production and proton leak. Changes in this basic parameter in patients may indicate altered or malfunctioning mitochondria [61]. Consistent with our results is another work where the authors observed a lower basal respiration rate and, consequently, reduced production in the mitochondria of ALS patients [62]. Ehinger et al., however, came to contradictory results in ALS patients. In their work, they observed a 36% increase in basal respiration and a 23% increase in maximal respiratory capacity compared to the control group, and they did not observe any significant differences in escape respiration. However, the authors described a major limitation of their study, the small sample size of patients [63]. 

Previous work has shown that complex I dysfunction significantly contributes to mitochondrial dysfunction in ALS. This dysfunction was also observed in our patient cohort. A decrease in complex I activity has also been demonstrated by the work of other authors [64,65,66,67]. The mitochondrial respiratory chain is responsible for producing energy in the form of ATP, and complex I is the first and largest enzyme in this chain, contributing to most of the proton motive force that drives ATP synthesis [68]. Inhibition of mitochondrial complex I in ALS has been known about since 1998 [49,69]. The role of complex I is to oxidize NADH and reduce ubiquinone to create part of the proton gradient required for ATP synthesis. Mutations in genes encoding complex I subunits have been found in ALS patients, and postmortem examinations have revealed reduced complex I activity in the motor neurons of ALS patients. In addition, animal models of ALS have shown that increasing complex I activity can improve motor function and prolong lifespan in these animals, suggesting that targeting complex I could be a potential treatment option for ALS [70]. Hor et al., in their study, demonstrated decreased mitochondrial respiration and increased glycolysis in ALS patients. Specifically, they observed reduced basal respiration, lower ATP production, and maximal respiratory capacity in patients [71]. 

The differences in maximal respiratory capacity that we also observed in our patients may signal dysfunction in respiratory complexes or cellular or mitochondrial substrate uptake/loading [72]. They then continued their study by creating isogenic cell lines; by activating SIRT3 and treating with nicotinamide, they observed a reversal of the defect in mitochondrial respiration [71]. Lastes et al., in their study, observed higher basal cell respiration in ALS patients than in the control group. However, reserve respiratory capacity, a parameter important in coping with increased energy demand, was significantly lower. If the reserve respiratory capacity is insufficient to provide enough ATP, it may indicate cell death and damage [73]. They also observed higher lactate production in ALS patients. An interesting observation was increased glycolytic capacity and decreased glycolytic reserve. The authors conclude that such mitochondrial damage would lead to increased ROS production and ATP production via the glycolysis pathway [74]. An increase in the production of reactive oxygen species was also observed in another study, which also showed depolarization of the mitochondria, impaired oxidative phosphorylation, decreased ATP production, and defective import of mitochondrial proteins [62]. 

In our work, we observed a decrease in mitochondrial respiration after adding digitonin when respiration is dependent on complex II. Complex II is an essential component of the Krebs cycle as well as the mitochondrial respiratory chain, both of which play an important role in ATP production [75]. As complex II is located at the junction of two key pathways, the Krebs cycle and oxidative phosphorylation, there is a suggestion that the function, regulation, and response of complex II to pathophysiological stimuli are key to the bioenergetics of the cell and the possible development of disease [76]. Previously, authors have shown that damaged complex II is capable of producing oxygen radicals [77]. Since most of the work focuses on complex I, we could not find any work to compare the results. An alternative interpretation of the digitonin effect is the hypersensitivity of the cells to the addition of digitonin, as the mitochondrial membrane underwent the first changes under apoptosis [78].

Defects in mitochondrial respiration have been implicated in various neurodegenerative diseases, such as Alzheimer’s disease and Parkinson’s disease. Studies focusing on Parkinson’s disease have observed unaltered basal respiration [79] and increased maximal respiratory capacity [79,80]. Shirinzi et al. also observed an increase in respiratory reserve [79]. Other studies have shown low activity of complex I [81,82] and low activities of complexes II and IV [82]. Work focused on Alzheimer’s disease has shown reductions in basal respiration and maximal respiratory capacity [83]. Other work has observed increased complex II and IV activity [84]. Frontotemporal lobar degeneration (FTLD) is a clinically and pathologically heterogeneous syndrome characterized by a progressive decline in behavior or language associated with degeneration of the frontal and anterior temporal lobes [85]. We were unable to find studies describing mitochondrial respiration in this disorder. Although some papers have focused on defects in mitochondrial respiration in individual diseases, there is no available evidence that examines differences between neurodegenerative diseases.

Available evidence suggests that at the intracellular level, mitochondria are the earliest targets in the pathophysiology of ALS. One expected consequence of impaired mitochondrial respiration is reduced ATP production and subsequent bioenergetic failure [86]. Another consequence of impaired mitochondrial respiration is increased reactive oxygen species (ROS) production. Under normal circumstances, the small amount of molecular oxygen in the mitochondria is reduced to ROSs (such as superoxide radicals) instead of being converted to water. However, in the normal state, these levels are minimal due to the antioxidants present in the mitochondria. According to the available knowledge, a disturbance of complex I will disrupt this homeostasis, and a dramatic increase in reactive oxygen species will occur [87]. One consequence is mitochondrial instability, which will trigger a negative cycle of increasing ROS production, damage, and mitochondrial malfunction. This can further damage mitochondria and lead to the release of mitochondrial proapoptotic proteins (cytochrome C, apoptosis-inducing factor) and ultimately to programmed cell death [88]. The result of this chain of events is neurodegeneration. Potential strategies to improve mitochondrial function include the use of dichloroacetate (the pyruvate dehydrogenase inhibitor DCA stimulates the conversion of pyruvate to acetyl coenzyme A, thereby supplying additional energy substrates to the TCA cycle) [89], ketogenic and high-fat diets, acetylcarnitine (plays a key role in the transport of long-chain fatty acids across mitochondrial membranes and limits the rate of β-oxidation [90]), and mitochondria-targeted antioxidants. In addition, antiapoptotic agents such as the mPTP-targeting agents minocycline and rasagiline are discussed [91]. In the future, it can be anticipated that novel ways of pharmacologically or genetically modulating mitochondrial turnover, movement, and dynamics in ALS, as well as restoring bioenergetic balance in the body, may become promising therapeutic strategies.

In summary, our data demonstrated impaired respiratory function in our group of patients with amyotrophic lateral sclerosis. We observed differences compared to healthy controls in basal respiration and impairment of complex I and complex II of the respiratory chain. 

High-resolution respirometry is an excellent means of assessing the control of mitochondrial respiration or studying cell oxygen kinetics in response to the delivery of specific electron-providing substrates, proton ionophores, and/or inhibitors of mitochondrial complexes. However, it should be mentioned that the study of amyotrophic sclerosis had limitations. Since this disease is rare, a significant limitation is the low number of patients. An overview of the limitations and ideas for further research are presented in Table 6.

## 5. Conclusions

Amyotrophic lateral sclerosis is a devastating neurodegenerative disease that is currently incurable. Although it is a rare disease, its incidence is expected to increase in the future. Data from preclinical, epidemiological, histopathological, and clinical studies indicate that mitochondrial dysfunction is a significant factor in the development of this disease. Current experimental evidence suggests that several mitochondrial pathways contribute to the pathogenesis of ALS. Our work demonstrates significant alterations in mitochondrial respiration in patients with amyotrophic lateral sclerosis. We observed decreased activity of complex I. An interesting finding was the reduced activity of complex II, as most studies focused their activity only on complex I. However, despite the overwhelming evidence suggesting that mitochondrial function is essential in the pathogenesis of ALS, the ability to identify the initial event in the cascade of changes that lead to neurodegeneration remains poorly understood. Therefore, future research should focus on investigating the mechanisms that lead to this damage. In addition, studying the interactions between mitochondrial respiration and other cellular processes involved in ALS, such as protein aggregation and oxidative stress, may also provide valuable insights into the mechanism of the disease. A better understanding of the involvement of mitochondria in the causes of this disease could lead to the discovery of appropriate diagnostic tools and, consequently, the development of new approaches to the therapy of this serious disease.

## Figures and Tables

**Table 1:** Overview of pathophysiological mechanisms underlying the development of ALS and potential use in therapy. ROS—reactive oxygen species, CNS—central nervous system, ER—endoplasmic reticulum, OPC—oligodendrocyte precursor cells, * drugs used in clinical practice.

Physiopathogenic Mechanisms	Involvement in the Pathophysiology	Method of Therapeutic Intervention	Drugs	Mechanism of Action
Genetic mutation	More than 120 genes linked to disease pathophysiology The most common mutations in genes: SOD1, FUS, TDP 43, C9ORF72, TARDBP		Tofersen	Antisense oligonucleotide against SOD1 [4]
		Antisense oligonucleotide therapy	ION-363	Antisense oligonucleotide against FUS [5]
			WVE-004	Antisense oligonucleotide against C9orf72 [6]
Oxidative stress	An initiating factor; the accumulation of ROS leads to cell damage and subsequent cell death due to an imbalance of radical generation and antioxidant protection	Antioxidants and modified oxidative stress	Verdiperstat	Myeloperoxidase inhibitor [7]
			RT001	Provides resistance to membrane lipid peroxidation [8]
Mitochondrial dysfunction	An early change; a key mechanism, ALS can promote mitochondrial fission and lead to fragmentation of the mitochondrial network through an increase in fission factors and a decrease in fusion factors. Electron transport chain dysfunction leads to higher mitochondrial oxygen consumption and ROS generation levels, reduced ATP synthesis, and DNA repair.	Mitochondria-targetted antioxidant therapy	Edavarone *	Antioxidant that removes oxygen radicals and eliminates lipid peroxides in the CNS [9]
			Sodium phenylbutyrate and Taurursodiol *	Decreases ER stress and mitochondrial dysfunction [10]
			SBT-272	Mitochondria-targeted novel peptide and peptidomimetic [11]
Microglia or macrophage	Infiltration of the gray and white matter of the spinal cord by macrophages surrounding the infiltrated nerve cells. Role of macrophages in causing neuroinflammation and subsequent degeneration of motor neurons	Anti-inflamatory therapy Autophagy targetted	NP001	Macrophage activation regulator [12]
Neuroinflammation	Activated microglia can activate astrocyte neurotoxicity by secreting inflammatory factors.	Modulation of the inflammatory environment	Masitinib Aldesleukin Pegcetacoplan	CSF1R kinase inhibitor [13] Recombinant human IL-2 [14] Complement C3 regulator [7]
Axonal transport defects	A likely factor, it is one of the earliest impairments seen in ALS, but its underlying cause remains unclear	Targeting microtubules and motor proteins	Noscapine	Stabilizes microtubules by binding to α-tubulin [15]
Oligodendrocyte dysfunction	Insufficient local energy supply from oligodendrocytes to axons leads to an energy deficit of motor neurons. Oxidative stress damage and an abnormal immune microenvironment lead to impaired oligodendrocyte differentiation, causing demyelination and degeneration of motor neurons.	Restoration of lipid homeostasis (remyelination) protection against oxidative stress, promotion of proliferation and maturation	Montelucast	Promotes OPC differentiation and remyelination in vivo [16]
			Bazedoxifene	Enhances myelin production by stimulating cholesterol biosynthesis in OL [17]
Nucleocytoplasmic defects	Impaired vesicle transport can cause protein aggregation and fragmentation of the Golgi apparatus	Down-regulation of exportin I	(SINE)-KPT-276	Selective inhibitor of nuclear export [18]
Glutamate excitotoxicity	Glutamate receptor hyperstimulation, increased synaptic glutamate release, altered AMPARs, decreased clearance of glutamate by astrocytes	Glutamate inhibitors improve motor function	Riluzole *	Glutamate antagonist that inhibits glutamate release and protein kinase C [19]
Impaired proteostasis	Misfolded proteins and protein aggregates accumulate in affected neurons and surrounding supporting cells	Increase in proteosome activity	Trehalose	Repurposed disaccharide that may prevent mutant protein aggregation [20]
			Trametinib	Mitogen-activated protein kinase (MEK) inhibitor that aims to activate the autophagy-lysosome pathway [21]

**Table 2:** Inclusion and exclusion criteria of ALS patients.

Inclusion Criteria	Exclusion Criteria
Adults age 18–80	Diagnosis of frontotemporal dementia or any other neurodegenerative diseases
Definitive diagnosis based on revised El Escorial criteria	Any acute or chronic condition that would limit the ability of the patient to participate in the study
Electromyographic findings meeting the Awaji Shima criteria	Unavailable family medical history
	Refusal to give informed consent

**Table 3:** Characteristics of patient cohort: diagnostic delay is the time from the appearance of the first symptoms to a definitive diagnosis; disease duration is the time from the first symptoms of the disease to the measurement of mitochondrial respiration; ΔFS ratio (rate of disease progression) is the rate of disease progression, ΔFS = (ALSFR-S total − ALSFR-S actual)/the duration of the disease from the onset of symptoms. Milgamma N-benfotiamine (40 mg), pyridoxinium chloride (90 mg), cyanocobalamin (0.25 g).

	Gender	Age	Diagnostic Delay (Months)	Duration (Month)	ALSFRS Score	Medication	Symptoms	Onset of Symptoms
1	Male	42	6	16	42	Riluzole Coenzyme Q10 Milgamma N	Left lower limb weakness, stiffness, motor problem, spastic-paretic gait	Spinal
2	Female	59	4	4	39	Riluzole Coenzyme Q10 Magnesium	Twitching in the right upper limb, twitching, weaker lower limbs	Spinal
3	Male	46	11	12	42	No medication	Numbness of the fingers on the upper limbs, twitching of the body, fatigue	Spinal
4	Male	44	8	21	30	Riluzole Coenzyme Q10 Milgamma N	Quadriparesis with dominant involvement of the left-sided limbs, the degree of paresis is most pronounced in the left upper limb	Spinal
5	Female	56	12	16	43	Riluzole Coenzyme Q10	Speech and swallowing problems, altered voice, progressive weakness of acral left upper limb, muscle hypotrophy, twitching of body muscles, progressively weaker left lower limb, slight shortness of breath on faster walking, exertion	Bulbar
6	Female	52	18	46	43	Riluzole Coenzyme Q10	Head drooping, PEG, anarthria	Bulbar
7	Male	46	9	77	38	Riluzole Coenzyme Q10 Milgamma N	Lower limb weakness, right upper limb weakness, wheelchair mobility	Spinal
8	Male	33	11	12	43	Riluzole Milgamma N	Tingling, progressive weakness of the left upper limb, posture, and gait normal	Spinal
9	Male	64	24	29	36	Riluzole Milgamma N	Upper and lower limb weakness, dysarthric speech, dysphagia, hemiparetic gait	Bulbar
10	Female	70	4	22	15	Riluzole Coenzyme Q10 Milgamma N	Spastic quadriparesis, dysarthria, wheelchair movement	Spinal
11	Male	80	24	24	35	No medication	Weakness and hypotrophy of the muscles of the right upper limb, gradual weakness also in the left upper limb, weakness of the lower limbs, impairment of speech and swallowing	Spinal
12	Female	62	53	63	37	No medication	Progressive quadriparesis, dysarthric speech, wheelchair movement	Spinal
13	Female	60	36	36	25	Riluzole	Mild dysarthria, bulbar syndrome, signs of mixed quadriparesis with advanced muscle hypotrophy, walking with the help of another person	Spinal
14	Female	56	16	17	41	No medication	Weakness of the right upper and lower limbs, progressive triparesis of the limbs	Spinal
15	Male	59	8	8	34	Riluzole Coenzyme Q10 Milgamma N	Quadriparesis, dysarthria, weakening of neck and axial muscles, present generalized fasciculations, impaired swallowing	Bulbar
16	Female	51	6	10	45	Riluzole Coenzyme Q10	Weakness of the lower limbs, instability when walking, weakness of the left upper limb	Spinal
17	Female	56	5	11	43	Riluzole Coenzyme Q10 Milgamma N	Dysarthria, difficulty swallowing, weakened neck muscles, muscle twitching	bulbar
18	Male	69	6	6	41	Riluzole Coenzyme Q10 Milgamma N	Weakness of the lower limbs, gradually also weakness of the left upper limb	Spinal
19	Male	37	4	4	37	No medication	Impaired leg mobility and buckling, upper limb insecurity and clumsiness	Spinal
20	Male	28	6	8	37	Riluzole	Weakness, clumsiness of the right upper limb, muscle twitching, impaired articulation	Spinal
21	Female	69	9	9	36	Milgama N	Lower limb weakness, gait impairment, knee buckling, upper limb weakness, walking with the aid of a cane	Spinal
22	Female	37	5	5	42	No medication	Weakness of the lower limbs, problems with walking	Spinal
23	Female	55	12	12	25	No medication	Upper limb weakness and stiffness acral left lower limb weakness	Spinal
24	Female	66	3	3	45	No medication	bulbar syndrome (dysarthria, dysphonia, dysphagia, discrete fasciculations on the tongue)	bulbar
25	Male	55	14	14	46	No medication	Progressive asymmetric weakness of the upper limbs	Spinal
26	Male	65	4	6	38	Riluzole Coenzyme Q10 Milgamma N	Weakness of the lower limbs, motor problem of the right upper limb, walking with the help of a cane	Spinal

**Table 4:** Inclusion and exclusion criteria of a control group.

Inclusion Criteria	Exclusion Criteria
Adults age 18–80	Occurrence of neurodegenerative diseases in the family history
Healthy with no history of chronic diseases	Any acute or chronic condition that would limit the ability of the patient to participate in the study
Without chronic treatment	Missing family history
persons without any family relation to the patients	Refusal to give informed consent

**Table 5:** Mean values with standard deviations of mitochondrial respiration in patients with amyotrophic lateral sclerosis and healthy controls.

State/Substrate	Control Subjects	ALS Patients
**Basal respiration**	26.42 ± 1.41	18.63 ± 2.77
**Pyruvate**	32.15 ± 1.76	22.74 ± 3.39
**Oligomycin**	4.77 ± 0.60	2.83 ± 1.62
**Uncoupler**	71.01 ± 4.31	51.02 ± 6.66
**Rotenone**	0.53 ± 0.50	0.64 ± 0.74
**Succinate**	5.70 ± 0.73	5.37 ± 0.91
**Digitonin**	58.25 ± 4.45	42.01 ± 6.17
**Cytochrome C**	57.94 ± 4.35	42.57 ± 6.30
**Antimycin A**	0.27 ± 0.14	0.01 ± 0.14

**Table 6:** Study limitations and suggestions for further research.

Limitations of the Study	Ideas for Future Research
Sporadic disease, limited number of patients Lack of studies addressing this disease	Studies on a larger number of patients Further work to validate the results obtained
Diagnostic delay	Exploring new biomarkers that could lead to earlier diagnosis of the disease
Heterogeneity of the disease	Monitoring interactions of pathophysiological mechanisms and risk factors
	Elucidation of mitochondrial respiration differences between neurodegenerative diseases
Individual rate of disease progression	Longitudinal follow-up of patients over time and assessment of changes according to the rate of disease progression

## Data Availability

The raw data supporting the conclusions of this article will be made available by the authors on request.
